# G protein-coupled receptors that influence lifespan of human and animal models

**DOI:** 10.1007/s10522-021-09945-8

**Published:** 2021-12-03

**Authors:** Francisco Alejandro Lagunas-Rangel

**Affiliations:** Department of Neuroscience, Functional Pharmacology, Uppsala University, Husargatan 3, BMC Box 593, 751 24 Uppsala, Sweden

**Keywords:** GPCR, Longevity, Healthspan, Dietary restriction, Insulin signaling, AMPK pathway

## Abstract

Humanity has always sought to live longer and for this, multiple strategies have been tried with varying results. In this sense, G protein-coupled receptors (GPCRs) may be a good option to try to prolong our life while maintaining good health since they have a substantial participation in a wide variety of processes of human pathophysiology and are one of the main therapeutic targets. In this way, we present the analysis of a series of GPCRs whose activity has been shown to affect the lifespan of animal and human models, and in which we put a special interest in describing the molecular mechanisms involved. Our compilation of data revealed that the mechanisms most involved in the role of GPCRs in lifespan are those that mimic dietary restriction, those related to insulin signaling and the AMPK and TOR pathways, and those that alter oxidative homeostasis and severe and/or chronic inflammation. We also discuss the possibility of using agonist or antagonist drugs, depending on the beneficial or harmful effects of each GPCR, in order to prolong people's lifespan and healthspan.

**Supplementary Information:**

The online version contains supplementary material available at 10.1007/s10522-021-09945-8.

## Introduction

One of humanity's oldest dreams has been to find a way to evade death for as long as possible. Indeed, this is one of the recurring themes used by fantasy, science fiction, and utopian novels. We have tried to investigate and obtain clues from those animals that have longer lives than ours, such as some species of whales, elephants and turtles (Austad 2010; Fushan et al. 2015; Lagunas-Rangel 2021), and also of those that are long-lived compared to other animals of similar size such as birds, bats, naked and blind mole-rats, among others (Gorbunova et al. 2014; Lagunas-Rangel and Chávez-Valencia 2017; Lagunas-Rangel 2018, 2020, 2021). A wide variety of branches of science work continuously, under different approaches and often together, to explore different ways of prolonging life, improving its quality and understanding the associated mechanisms, and although much remains to be investigated, in recent decades there have been substantial advances in this area (Bayersdorf and Schumacher 2019). Lifespan is defined as the maximum number of years that an organism can live, while life expectancy is the average total number of years that an organism achieves (Tosato et al. 2007). Meanwhile, healthspan is the number of years that an organism has a good general state of health without serious diseases, disabilities or chronic diseases related to age (Kaeberlein 2018). Longevity is generally defined as a long duration of life and is the result of a decrease in cumulative mortality in any population across all ages (Karam Singh and Watson 2014; Gu et al. 2015).

There are many factors that determine how long a person will live, including behavior, diet, physical activity, sanitation, living conditions, medical care, genetic, immune, and environmental factors (Zhang et al. 2020). Different medical and technological advances have allowed life expectancy to increase a little more each time. This is clearly seen if we take into account that in the early nineteenth century no country in the world had a life expectancy greater than 40 years, in 1950 the global average life expectancy was 46 years, in 2000 it was 66.6 years, in 2015 it was 71 years and by 2019 it was 73.4 years (Riley 2005; Roser et al. 2013; WHO 2020).

Previously, genetic and pharmacological studies in model organisms (yeast, worms, flies and rodents) identified multiple means of prolonging lifespan and healthspan, highlighting dietary restriction (Fontana et al. 2010), inactivation of the insulin receptor (Bluher 2003) or insulin-like growth factor 1 receptor (IGF1R) (Holzenberger et al. 2003), knockout of the growth hormone receptor (GHR) (Coschigano et al. 2000), anterior pituitary gland impairment (Bartke and Brown-Borg 2004), inhibition of the mechanistic target of rapamycin (mTOR) pathway (Harrison et al. 2009; Selman et al. 2009), activation of 5' adenosine monophosphate-activated protein kinase (AMPK) (Martin-Montalvo et al. 2013), increased autophagy (Pyo et al. 2013), the overexpression of certain sirtuin proteins (Kanfi et al. 2012; Satoh et al. 2013; Lagunas-Rangel 2019; Lagunas‐Rangel et al. 2021), the overexpression of the fibroblast growth factor 21 (FGF-21) (Mendelsohn and Larrick 2012), and the deactivation of the pain receptor TRPV1 (Riera et al. 2014), among others. However, despite these advances, the question remains whether these mechanisms can be extrapolated to humans and whether existing or new drugs or therapies can be used to promote increased and healthy lifespan.

G protein-coupled receptors (GPCRs) are one of the main groups of proteins used as drug targets, mainly because they comprise the largest superfamily of proteins in mammalian genomes (~ 800 members), they possess binding sites to drugs that are accessible on the cell surface and notably mediate most of the cellular responses to external stimuli such as light, odors, nutrients, hormones, neurotransmitters, and growth factors, and therefore have a fundamental role in human pathophysiology, both normal and pathological (Katritch et al. 2013; Hauser et al. 2017; Sriram and Insel 2018). In this sense, the overexpression or deletion of different GPCRs in animal study models have allowed them to have a longer lifespan compared to their wild counterparts and, on many occasions, they also acquire greater resistance to a variety of stressful conditions such as starvation, environments with temperatures higher than optimal and presence of oxidizing agents (Table 1) (Taormina et al. 2019). Furthermore, epidemiological and genetic studies have shown that certain single nucleotide polymorphisms (SNPs) that modify the activity or expression of GPCRs appear more frequently in people with extreme longevity (centenarians or supercentenarians) (Table 1) (Campa et al. 2012; Corbo et al. 2013; Benigni et al. 2013; Crocco et al. 2016). All these findings together with the fact that currently the activity of a large number of GPCRs is pharmacologically controllable with the use of agonist or antagonist drugs (Lagerström and Schiöth 2008; Hauser et al. 2017), makes them potential targets to prolong our health and life. However, although GPCRs have been extensively studied in various respects (Vassart and Costagliola 2011; Wang et al. 2019), their role in longevity is not fully understood and most of the findings on this topic have been indirect.

**Table 1:** GPCRs that have shown effects on lifespan

GPCR name	Organism	Role in extending lifespan	Effect associated with lifespan	References
TAS2R16	*H. sapiens*	Favors	Alters recognition of beneficial and harmful molecules	Di Bona et al. (2020), Malovini et al. (2019), Campa et al. (2012)
TAS2R38	*H. sapiens*	Favors	Alters recognition of beneficial and harmful molecules	Melis et al. (2019)
HTR2A	*H. sapiens*	Favors	Associated with schizophrenia, suicide, impulsivity, and emotional disorders	Jobim et al. (2008), Fukuda et al. (2006)
DRD2	*H. sapiens*	Favors	–	Crocco et al. (2016), Szekely et al. (2016)
AGTR2	*M. musculus*	Favors	Participates in processes of brain damage and proteinuria	Gelosa et al. (2009)
ADRA1A	*M. musculus*	Favors	–	Doze et al. (2011)
AdipoR1	*M. musculus*	Favors	Regulates the AMPK pathway and the CaMKKb-AMPK-SIRT1 axis	Iwabu et al. (2010), Yamauchi et al. (2007)
AdipoR2	*M. musculus*	Favors	Changes the expression of PPAR-α target genes. Alters fatty acid oxidation and pro-inflammatory cytokine levels	Yamauchi et al. (2007)
HCRTR2	*M. musculus*	Favors	Alters neuronal activity and its beneficial effects	Satoh et al. (2013)
GPBAR1	*M. musculus*	Favors	Controls mechanisms that mimic dietary restriction	Wang et al. (2017a, b)
DOP-3	*C. elegans*	Favors	Controls the release of acetylcholine	Saharia et al. (2016)
STR-2	*C. elegans*	Favors	Required at temperatures ≥ 20 °C. Regulates lipid droplet homeostasis and lipid metabolism	Dixit et al. (2020)
SRBC-48	*C. elegans*	Favors	Protects against infection-associated dendritic degeneration. Avoid uncontrolled activation of immune genes	Kaur and Aballay (2020)
DAF-37	*C. elegans*	Favors	Regulates Sir2.1-dependent mechanisms	Ludewig et al. (2013)
mGluR	*D. melanogaster*	Favors	Controls increases in calcium mediated by the ionotropic glutamate receptor and possibly GH secretion	Ly and Naidoo 2019)
TkR99D	*D. melanogaster*	Favors	Alters Dilp2 and Dilp3 secretion	Birse et al. (2011)
BOSS	*D. melanogaster*	Favors	Alters oxygen homeostasis	Kohyama-Koganeya et al. (2017)
CapaR	*D. melanogaster*	Favors	Inhibits the release of glucagon-like adipokinetic hormone from the cardiac body Restricts the energy mobilization of adipose tissue and prevents harmful hyperglycemia	Koyama et al. (2021)
ADRB2	*H. sapiens*	Disadvantages	–	Zhao et al. (2012), Gao et al. (2003), Spindler et al. (2013)
FSH	*H. sapiens*	Disadvantages	Regulates fertility, activation of BAT and energy expenditure	Corbo et al. (2013) Liu et al. (2017)
AGTR1	*M. musculus* *H. sapiens*	Disadvantages	Alters oxidative homeostasis and cardiac and endothelial function. Causes changes in the levels of NAMPT and SIRT3	Benigni et al. (2013), Mercier et al. (2007), Nikiforovich et al. (2006), Nagura et al. (1995)
ADRA1B	*M. musculus*	Disadvantages	–	Collette et al. (2014)
SER-1	*C. elegans*	Disadvantages	Alters resistance to stressors	Murakami and Murakami (2007)
SER-3	*C. elegans*	Disadvantages	Participates in mechanisms that mimic dietary restriction	Zhang et al. (2021), Petrascheck et al. (2007)
NPR1	*C. elegans*	Disadvantages	Alters oxygen homeostasis	Abergel et al. (2017)
GAR-3	*C. elegans*	Disadvantages	Alters acetylcholine signaling	Lucanic et al. (2016)
OCTR-1	*C. elegans*	Disadvantages	Controls the expression of several immune defense genes	Wibisono et al. (2021)
OR83B	*D. melanogaster*	Disadvantages	Alters resistance to stressors. Controls mechanisms that mimic dietary restriction	Libert et al. (2007)
CCHa2R	*D. melanogaster*	Disadvantages	Regulates insulin release	Jin et al. (2020)
Mth	*D. melanogaster*	Disadvantages	Alters resistance to stressors. Regulates the levels of insulin released and the expression of SODs	Gimenez et al. (2013), Lin (1998)
Mthl10	*D. melanogaster*	Disadvantages	Alters Dilp2 secretion and metabolic inflammation	Sung et al. (2017)

GPCRs can provide beneficial or harmful effects and therefore affect the lifespan of organisms. The aforementioned organism is where GPCR has been studied the most

In this way, the objective of this review is to discuss how the signaling of different GPCRs affects the lifespan of various study models and attempts to glimpse the associated molecular mechanisms that could be extrapolated in humans. We also highlight the possibility of using current and new drugs and/or therapies targeting specific GPCRs in order to improve lifespan and healthspan.

## GPCR signaling

Structurally, GPCRs are integral proteins that are characterized by crossing the cell membrane seven times, almost always with an extracellular N-terminus and an intracellular C-terminus, and most exhibit a basal level of activity in the absence of a ligand (Sriram and Insel 2018). Based on their sequence and evolutionary conservation, these receptors are divided into subfamilies that include class A (similar to rhodopsin), class B1 (similar to the secretin receptor), class B2 (adhesion receptors), class C (similar to the metabotropic glutamate receptor) and class F (frizzled type) as well as a class T corresponding to a taste 2 sensory receptor subfamily (Wootten et al. 2018).

Overall, when a ligand binds to a GPCR, it undergoes a conformational change that allows it to interact with a heterotrimeric G protein (composed of alpha (α), beta (β) and gamma (γ) subunits) and act as a guanine nucleotide exchange factor (GEF) that facilitates the exchange of guanosine diphosphate (GDP) for guanosine triphosphate (GTP) in the Gα subunit. In humans, there are 16 Gα, 5 Gβ, and 13 Gγ subunits that can combine to form a wide range of heterotrimeric G proteins (Nelson and Cox 2014; Weis and Kobilka 2018; Wootten et al. 2018). GTP bound to Gα triggers the dissociation of the Gα subunit from the Gβγ dimer and from the receptor. The dissociated Gα and Gβγ subunits interact with other intracellular proteins to continue the signal transduction cascade, while the released GPCR can reattach another heterotrimeric G protein to form a new complex that is ready to initiate another round of signal transduction (Nelson and Cox 2014; Weis and Kobilka 2018; Wu et al. 2019). Furthermore, GPCRs can activate other signaling pathways through arrestins, which deactivate G protein signaling by preventing its interaction with the receptor and promoting the internalization of the latter (Nelson and Cox 2014; Weis and Kobilka 2018).

The signaling pathways stimulated by each GPCR depend on the associated Gα subunit that can be subdivided into four families (based on sequence similarity) which are Gα_s_, Gα_i_, Gα_q/11_ and Gα_12/13_. Gα_s_ family activates adenylyl cyclases to catalyze the conversion of adenosine triphosphate (ATP) to cyclic adenosine monophosphate (cAMP) and thereby activate protein kinase A (PKA), while Gα_i_ family primarily inhibits cAMP production, activates a variety of phospholipases and phosphodiesterases, and promotes the opening of various ion channels. The Gα_q/11_ family activates isoform β of the phospholipase C (PLC) enzyme to convert phosphatidylinositol 4,5-bisphosphate (PIP_2_) to diacylglycerol (DAG) and inositol 1,4,5-trisphosphate (IP_3_), which subsequently activate protein kinase C (PKC) and elevate intracellular Ca^2+^ levels. Finally, the Gα_12/13_ family associates with RhoGEF and stimulates the activity of Rho GTPases, resulting in the activation of multiple kinase cascades such as Rho and RAS-regulated kinases, mitogen-activated protein kinases (MAPK) and kinases regulated by second messengers (Nelson and Cox 2014; Wu et al. 2019).

## GPCR influencing lifespan

### Odorant receptors

Odorant receptor 83B (OR83B) is a GPCR with inverted membrane topology (intracellular N-terminus and extracellular C-terminus) that heterodimerizes with any of the 61 conventional odor receptors expressed on *Drosophila* olfactory sensory neurons and acts as an essential co-receptor for odor recognition (Larsson et al. 2004; Benton et al. 2006). OR83B promotes the trafficking of other olfactory receptors to dendrites, and apparently when these receptors are stimulated with an odor or pheromone, OR83B acts as an ion channel that causes the influx of Na^1+^/Ca^2+^ and the depolarization of the membrane (Larsson et al. 2004; Wicher et al. 2009). Loss-of-function mutations in the *Drosophila* OR83B increase the survival of flies, mainly in females, and cause an increase in triglyceride levels, the main lipid-storage molecule, that is independent of metabolic rate. Furthermore, flies with mutations also improved their resistance to hyperoxia and starvation compared to wild controls (Libert et al. 2007). Prolongation of life due to loss of OR83B is independent of insulin signaling (Libert et al. 2007), but the resulting decreased odor perception could influence dietary restriction-mediated longevity as occurs with *Caenorhabditis* (See section “Serotonin receptors”) (Alcedo and Kenyon 2004; Zhang et al. 2021). Endurance exercise in wild flies caused a decrease in the expression of this protein and the OR83B-mutated flies were able to run longer before being exhausted, their resistance to cardiac stress was greater and they preserved their climbing index until older ages than the controls (Sujkowski et al. 2015). This could indicate a feedback mechanism between the perception of odors and the beneficial effects of exercise, a phenomenon that has also been seen in humans (Schubert et al. 2013; Sollai and Crnjar 2021).

### Taste receptors

Extremely long-lived individuals, such as centenarians, make up only a small proportion (~ 0.01 to 0.02%) of the population, but their genes contain a biological blueprint for healthy aging and longevity (Zhang et al. 2020). Evidence has been presented that people who have a pair of functional alleles (PAV/PAV) in the taste receptor type 2 member 38 (TAS2R38) gene may have a favorable genetic status compared to people with non-functional alleles (AVI/AVI) to achieve greater longevity (Melis et al. 2019). Something similar was found with the single nucleotide polymorphism (SNP) rs978739 in taste receptor type 2 member 16 (TAS2R16), where the frequency of the homozygous A/A allele is higher in centenarians than the G/G allele (Campa et al. 2012; Malovini et al. 2019; Di Bona et al. 2020). In both cases, the increase in longevity could be due to a better recognition of the beneficial and harmful molecules that would guide the diet in a better direction such as the consumption of less fat, but also participate in other physiological functions such as efficient immune response and favorable body composition, among other things (Tepper et al. 2008; Lee and Cohen 2015; Smail 2019).

### Neuropeptide CCHamide-2 receptor (CCHa2R)

CCHa2R participates in the *Drosophila* fat body-brain axis to couple growth with nutritional status. This receptor is expressed at high levels in the insulin-producing cells (IPCs) of the fly brain, and by binding to its ligand, a small peptide called CCHamide-2 (CCHa2) that is expressed in the fat body and gut and whose expression is sensitive to the presence of nutrients (particularly sugars), leads to the secretion of *Drosophila* insulin-like peptides (Dilps) (Sano et al. 2015). A SNP in an intron of the gene encoding CCHa2R (identified as 2R_1939249_SNP and where a cytosine is exchanged for a thymine) was related to a decrease in the levels of metabolites such as serine, isoleucine, valine, methionine, and pipecolate, as well as increased survival in response to dietary restriction (Jin et al. 2020). SNPs in introns can affect mRNA stability, change the efficiency of its processing, and introduce or remove splice sites that result in truncated or non-functional proteins (Ward and Cooper 2010). Thus, this could indicate that this SNP causes a decrease in the levels of functional CCHa2R and correlates well with what was observed in flies where the expression of this gene was silenced, which showed an increase in the average lifespan under dietary restriction but not with an ad libitum diet (Jin et al. 2020). CCHa2R is associated with lifespan possibly due to its influence on metabolism. When the levels of this protein fall, it is likely that there is less insulin release from the IPCs, which, as already mentioned, is associated with a longer life span. Interestingly, the closest mammalian homologue of CCHa2R is bombesin-like receptor 3 (BRS3), an orphan GPCR that regulates food intake, metabolic rate, body temperature, heart rate, blood pressure, and insulin secretion (Xiao and Reitman 2016). BRS-3 agonists in mice have been shown to cause brown adipose tissue (BAT) activation with a persistent increase in metabolic rate, although their effect on lowering glucose levels was small, and have not been investigated its effects on life expectancy (Ohki-Hamazaki et al. 1997).

### Methuselah (Mth) and methuselah-like (Mthl)

The Mth/Mthl gene family arose in early metazoans and, as evolution progressed, underwent numerous extinction and expansion events that made it abundant in insects but absent in vertebrates (Patel et al. 2012; de Mendoza et al. 2016; Friedrich et al. 2018). Phylogenetic analyzes of Mth protein have shown that it has undergone an unusually high level of adaptive amino acid divergence concentrated in the intra- and extracellular loop domains as an evolutionary strategy in a signal transduction pathway that can modulate lifespan in nature (Schmidt et al. 2000). In addition, there was a strong latitudinal cline in the frequency of the most common haplotype that provides evidence of climatic selection operating this locus, with reduced haplotype diversity in northern insect populations from North American relative to southern populations (Schmidt et al. 2000; Duvernell et al. 2003). *Drosophila* has 15 paralogs of Mth that are identified as Mthl1-15 and which also have orthologs in other arthropods such as *Anopheles gambiae* (7), *Bombyx mori* (4), *Apis mellifera* (4), *Acyrthosiphon pisum* (3) and *Tribolium castaneum* (5), as well as non-arthropod species such as mollusks, hemichordates, and cnidarians (Hill et al. 2002; Fan et al. 2010; Bai et al. 2011; Li et al. 2013a, b; de Mendoza et al. 2016).

**Fig. 1:**
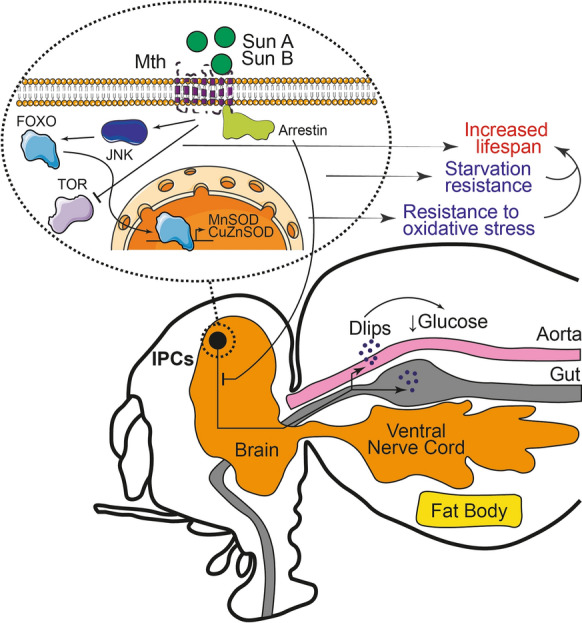
Disruption of Mth causes an increase in the lifespan of *Drosophila*. When Mth levels are reduced in IPCs, the secretion of Dlips is inhibited with a consequent increase in glucose levels in the hemolymph that protects against starvatation. JNK is also promoted to activate FOXO, which translocates to the nucleus where it promotes the transcription of MnSODs and CuZnSODs to increase resistance to oxidative stress and shelf life. TOR activity is inhibited to promote increased lifespan

Loss-of-function mutations in the *Drosophila* Mth receptor were shown to increase the survival of flies under conventional growth conditions, when fed paraquat (which induces oxidative stress), under starvation conditions, and when flies are kept at temperatures above 35 °C (Lin 1998). Indeed, the decrease in Mth expression only in IPCs was sufficient to increase longevity and resistance to oxidative stress (Gimenez et al. 2013). Although these properties appear to vary as a function of allelic variation, growth temperature (the differences were more notable at 29 °C), the age at which the stressor is administered (the older the age, the more differences), sex and mating (most noticeable in unpaired flies) (Mockett and Sohal 2006; Baldal et al. 2006; Paaby and Schmidt 2008). Similarly, N-terminal mutations in Sun A and Sun B peptides, the main identified endogenous Mth ligands (although the receptor has been shown to be promiscuous to different peptides), increased the lifespan of flies. Sun A and Sun B are encoded by the *stunted* gene and differ only in their size and in the sequence of the last amino acids (Cvejic et al. 2004). Flies' lifespan was also increased by constitutive expression of peptides with a highly conserved consensus sequence [R/P]XXWXXR (RWR motif) that bind to Mth and inhibit its signaling (Ja et al. 2007). Interestingly, it has been reported that Mth levels decrease when flies are fed for several days with some plant-based extracts such as apple, cranberry, blueberry, rosemary, and black rice (Peng et al. 2011, 2012; Zuo et al. 2012; Wang et al. 2015b, 2017a). It was previously reported that Mth and its Sun ligand act upstream to regulate the secretion of Dilps in response to nutrients, particularly the secretion of Dilp2 (Delanoue et al. 2016). Thus, the decrease in Mth expression in IPCs causes an inhibition of insulin secretion with the consequent increase in glucose in the hemolymph (Gimenez et al. 2013). Furthermore, it was also revealed that Mth silencing causes an increase in the activity of Mn- and Cu/Zn-superoxide dismutases (MnSOD and Cu/ZnSOD) (Gimenez et al. 2013). Experimental results have shown that both mechanisms alone can prolong the life of *Drosophila* (Aigaki et al. 2002) and, in this case, possibly occur in response to FOXO signaling and with likely JNK intervention (Gimenez et al. 2013). Also, it was found that TOR pathway, β-arrestin expression and FOXO levels translocating to the nucleus are important in the regulation of Mth-mediated longevity (Fig. 1) (Gimenez et al. 2013; Wang et al. 2015a).

On the other hand, it was shown that the Mthl10 deletion in *Drosophila* causes a phenomenon similar to that observed with dietary restriction and significantly increases its survival, mainly in females, and this was associated with a decrease in the secretion of Dilp2 from IPCs and a decrease in the metabolic inflammation, although it also favors its infection by pathogenic bacteria (Sung et al. 2017). However, Mthl proteins in other insects have shown opposite effects than expected. For example, the down regulation of the Mthl proteins of *Tribolium castaneum* decreased their survival and affected their resistance to starvation and heat, although for Mthl4 and Mthl5 their resistance to oxidative stress improved and for Mthl1 and Mthl2 their fertility decreased (Li et al. 2014b). Particularly for Mthl1, its effects on survival are thought to be due to a suppression of the Toll and immunodeficiency (IMD) pathways (Li et al. 2014a). The decrease in Mthl1 levels in *Lymantria dispar* made it more sensitive to the insecticide deltamethrin with a decreased expression of stress response genes such as heat shock proteins (HSP), some cytochrome P450 isoenzymes and glutathione S-transferases (GST), while its ectopic expression in flies increased its lifespan (Cao et al. 2019). In *Dastarcus helophoroides*, Mthl1 and Mthl5 increase their expression with the age of the insect, Mthl2 and Mtl5 increased their expression against oxidative stress, high growth temperature and starvation, mainly in females. Meanwhile, Mthl1 decreased with oxidative stress and did not have consistent changes with increasing temperature and starvation (Zhang et al. 2016).

### Serotonin receptors

The neurotransmitter serotonin is known to affect longevity variation in natural *Drosophila* populations and age-related changes in serotonin systems are known risk factors for age-related diseases in mammals (De Luca et al. 2003; Fidalgo et al. 2013). Dietary options have been reported to reduce life expectancy in *Drosophila*, and the 5HT2a receptor is one of the main culprits due to its role in establishing the protein value of meals (Ro et al. 2016). In this way, the interruption of their signaling eliminated the mortality differences between the two dietary environments and a change in the state of metabolic networks was observed throughout the organism from a fragmented, fragile and vulnerable to perturbations to a highly connected and robust one (Lyu et al. 2021b, a). Notably, experimental trials with *Caenorhabditis* found that mutations affecting the enzymes that regulate its biosynthesis and serotonin transporters produce small or modest effects on its lifespan. However, mutations in the receptors produced significant changes in their longevity (Murakami and Murakami 2007). Although the mechanism was not determined, loss-of-function mutations in *Caenorhabditis* serotonin receptor SER-1, which is similar to HTR2A and HTR2C in humans, prolonged the mean lifespan by up to 46% and maximum lifespan by 41%. This possibly associated with a better response to stressors such as temperature (35 °C) and UV light (Murakami and Murakami 2007). Mianserin, an antidepressant, has also been reported to increase the lifespan of this worm by inhibiting SER-3 and SER-4, which creates a "perceived" state of starvation (Petrascheck et al. 2007). In this sense, SER-3 has also been linked to the effect of food odors on dietary restriction-mediated longevity. In this circuit, ODR-3 expressing ADF neurons function as primary sensory neurons and, upon detecting any food odor, release serotonin which is taken up by SER-5 in CEP neurons. These neurons in turn release dopamine which stimulates DOP-6 and inhibits RIC neurons, thus preventing the release of octopamine and the activation of SER-3 and the downstream PLCβ-CaMKK-AMPK axis in the gut that have been implicated with increased longevity (Zhang et al. 2021). In this signaling, IFE-2 collaborates in an additive and synergistic way (Matei et al. 2021). In response to glutamate released by IL neurons at low temperatures, overexpression of MGL-1 in *Caenorhabditis* serotonergic NSM neurons promotes longer life expectancy through activation of SER-7 in the gut and activation of the transcription factor DAF -16 (Zhang et al. 2018). Meanwhile, people homozygous for the T allele of the SNP rs6313 in the HTR2A gene apparently live longer than people homozygous for the C allele or heterozygous. C allele of this SNP is thought to reduce receptor expression and has been linked to schizophrenia, suicide, impulsivity, and emotional disturbances (Fukuda et al. 2006; Jobim et al. 2008). The loss of the presynaptic receptor HTR1B significantly decreased the survival of mice and caused an early age-related motor impairment and the development of a genetic expression signature characteristic of aging (Sibille et al. 2007).

### Dopamine receptors

As with the serotonin system, the loss of dopamine activity with age is well documented. The dopamine system exhibits age-related declines both presynaptically and postsynaptically, thus decreasing the number of dopamine-containing neurons and the density of dopamine receptors (Morgan et al. 1987; Volkow et al. 2000). DOP-3 was shown to participate in the extension of the lifespan of *Caenorhabditis* when treated with the antihypertensive drug reserpine. DOP-3 signaling is known to inhibit acetylcholine release and the mechanism used by reserpine prolongs life by modulating the release of neurotransmitters, especially acetylcholine (Saharia et al. 2016). Likewise, ursolic acid showed that, at least partially, through the dopamine receptor DOP1 and to a lesser extent through DOP3, it reduces stress and prolongs the life of the worms (Naß and Efferth 2021). In *Drosophila*, the DRD2-like protein was identified as one of the main candidates mediating the increase in lifespan by the progesterone antagonist mifepristone (Landis et al. 2015). In addition, normal levels of the dopamine D2 receptor (DRD2) together with an enriched environment positively influence the life expectancy of mice, as does chronic treatment with selegiline, a monoamine oxidase inhibitor that increases dopamine levels in the brain (Knoll 1988; Thanos et al. 2016). A negative association was also observed between human longevity and the presence of the minor variant G of the SNP rs6276, mainly when the allele is homozygous, and this is explained because said SNP causes the DRD2 mRNA to degrade more rapidly when enhance its binding to various miRNAs such as miR-485-5p (Crocco et al. 2016). Meanwhile, a variable number tandem repeat (VNTR) has been reported in the third exon of the DRD4 gene and the seven-repeat allele was associated with increased levels of physical activity and longevity, preferably in women (Grady et al. 2013; Szekely et al. 2016).

### Angiotensin II receptors

In rodents, there is strong evidence that chronic antagonism of the renin-angiotensin system (RAS) prolongs life (Thornton 2011). Mice lacking angiotensin II receptor type 1 (AGTR1) increased their lifespan compared to their wild-type littermates, and exhibited less cardiac and vascular damage with age. It was observed that AGTR1 deficiency caused an increase in NAMPT and SIRT3 levels in mice, with reduction of oxidative damage and age-induced mitochondrial loss was avoided (Benigni et al. 2009). SIRT1 showed a reciprocal regulation with AGTR1, so that at high levels of SIRT1 the expression of AGTR1 decreased (Diaz-Ruiz et al. 2015). Indeed, resveratrol, a polyphenol activator of SIRT1 and which has been shown to prolong lifespan of several species, inhibits the expression of AGTR1 at the transcriptional level (Valenzano et al. 2006; Miyazaki et al. 2008; Lagunas-Rangel and Bermúdez-Cruz 2020). Previously, a longer survival of hypertensive rats was observed with long-term treatment using the AGTR1 blockers enalapril, losartan, valsartan and ME3221 (Nagura et al. 1995; Mercier et al. 2007). Similarly, long-term treatment with fonsartan doubles the lifespan of hypertensive rats by improving cardiac and endothelial function through increasing the expression of endothelial nitric oxide synthase (eNOS) in the heart and the carotid artery and decrease the expression and activity of angiotensin-converting enzyme (ACE) in tissues (Linz et al. 2000). In centenarians, a high frequency of the N298S mutation was found in the AGTR1 transmembrane helix, which has been reported to cause a reduction in binding sites to other proteins and an alteration in receptor signaling with respect to the wild-type (Nikiforovich et al. 2006; Benigni et al. 2013). In addition, some SNPs were identified in the AGTR1 gene associated with extreme longevity, highlighting rs422858 in the promoter region. rs422858 is a dinucleotide switch in which an AG (major allele) is exchanged for a CC (minor allele), and it has been suggested that it could affect the transcription of this gene. Centenarians homozygous for the minor allele rs275653 had fewer peripheral blood polymorphonuclear cells positive for AGTR1 and blood pressure (Benigni et al. 2013). Interestingly, a high dose of an angiotensin II receptor type 2 (AGTR2) agonist also increased the survival of hypertensive rats, delaying the onset of brain damage and proteinuria (Gelosa et al. 2009). Furthermore, growth hormone receptor (GHR) knockout mice, which survived significantly longer than their wild counterparts, showed reduced AGTR1 levels and increased AGTR2 levels (Giani et al. 2012). All of this could tell us about the opposite role of these two receptors in achieving a long life.

### Adrenergic receptors

Different adrenergic receptor subtypes have also shown opposite effects on survival (Collette et al. 2014). Mice expressing a constitutively active mutant alpha-1A adrenergic receptor (ADRA1A) lived significantly longer and had cardio- and neuroprotective effects and a lower incidence of cancer compared to wild animals, while mice expressing a constitutively active mutant alpha-1B adrenergic receptor (ADRA1B) lived significantly less time and had no changes in the incidence of cancer (Doze et al. 2011; Collette et al. 2014). Longevity has also been associated with beta-2 adrenergic receptor (ADRB2) haplotypes, which affect its translation efficiency. The AC haplotype consisting of two SNPs, rs1042718 and rs1042719, has been reported to reduce the expression of ADRB2 and was positively associated with male longevity, while the CG haplotype increased the translational efficiency of ADRB2 and was negatively associated with its longevity (Zhao et al. 2012). Coupled with this, transgenic mice which overexpress ADRB2 in heart tissue had a shorter lifespan and the males showed a more noticeable affectation (Gao et al. 2003). Furthermore, chronic treatment with the β -blockers metoprolol and nebivolol increased the lifespan of male mice and flies (Spindler et al. 2013).

### Adiponectin receptors (AdipoRs)

**Fig. 2:**
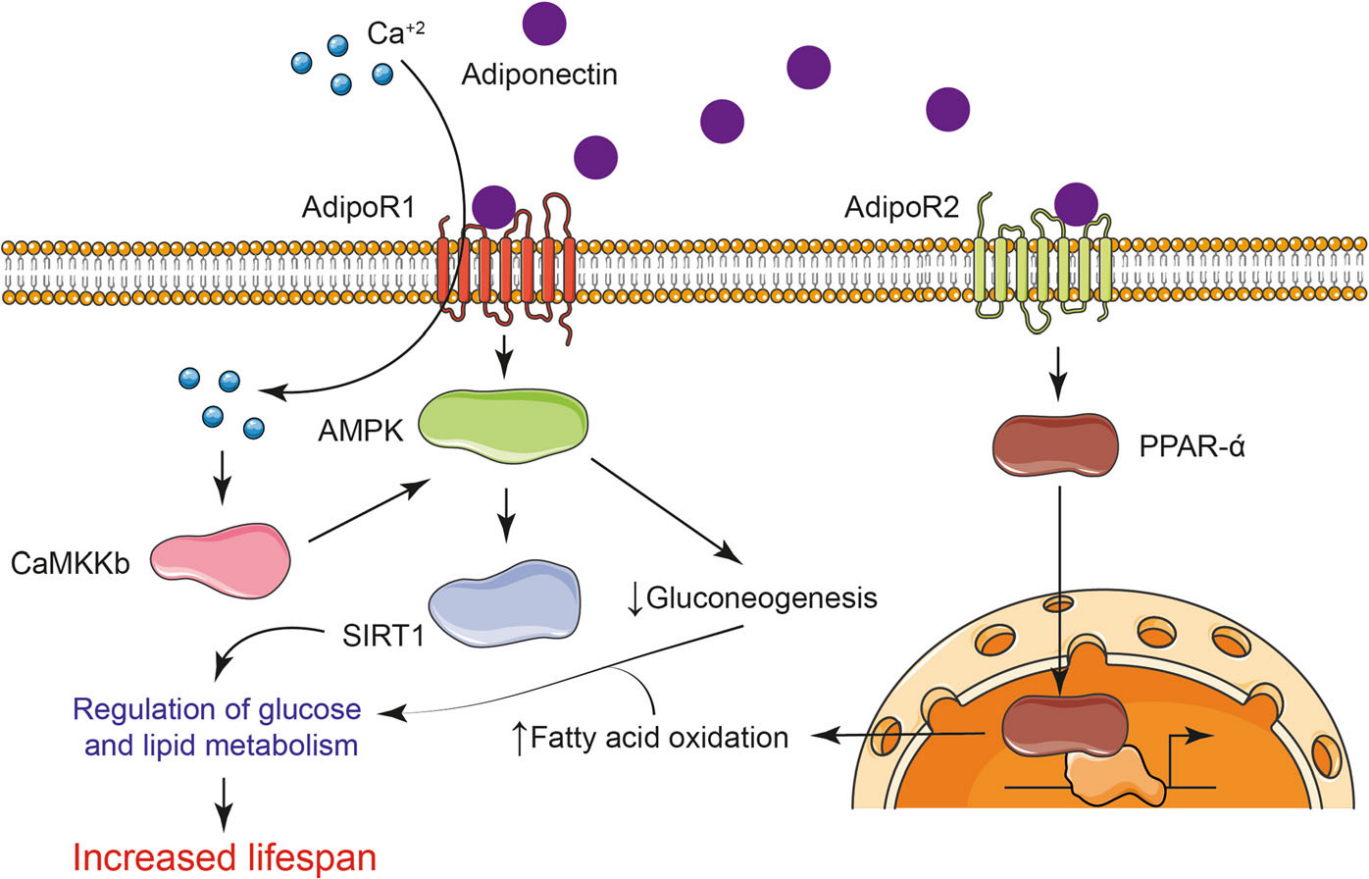
AdipoRs cooperate to improve lifespan and healthspan, AdipoR1 and AdipoR2 serve as adiponectin receptors and mediate the activation of the AMPK and PPAR pathways, thus regulating glucose and lipid metabolism that cooperates in increasing lifespan

Adiponectin is a hormone secreted by adipocytes whose reduction causes insulin resistance, glucose intolerance, dyslipidemia, and hypertension, and is strongly associated with such relevant diseases as obesity, diabetes, metabolic syndrome, and atherosclerosis (Yamauchi and Kadowaki 2013). In this sense, the adiponectin receptors AdipoR1 and AdipoR2 (which, like OR83B, have an inverted membrane topology) are decreased in animal models of obesity and type 2 diabetes (Yamauchi et al. 2007; Okada-Iwabu et al. 2015). Similar to calorie restriction and exercise, activating AdipoRs may have the potential to not only improve lifestyle-related illnesses, but to help extend shorter lifespan on an unhealthy high-calorie diet (Okada-Iwabu et al. 2015). Thus, the overexpression of AdipoR1 in the liver of a mouse model of human obesity caused an increase in the activation of the AMPK pathway, while the overexpression of AdipoR2 caused an increase in the expression of peroxisome proliferator activated receptor alpha (PPAR-α) target genes. AMPK activation reduced gluconeogenesis, while PPAR-α activity in both cases increased fatty acid oxidation and led to an improvement in diabetes (Yamauchi et al. 2007), and both mechanisms also contribute to increased longevity in animal models (Fig. 2) (Erol 2007; Martin-Montalvo et al. 2013). Furthermore, it has also been reported that AdipoR1 induces an extracellular influx of Ca^2+^ that is necessary for the subsequent activation of Ca^2+^/calmodulin-dependent protein kinase kinase b (CaMKKb), AMPK and SIRT1, increased expression and decreased acetylation of peroxisome proliferator-activated receptor C coactivator-1a (PGC-1a) and increased mitochondria in myocytes (Iwabu et al. 2010). Mice deficient in AdipoR1 or AdipoR2 and given a high-fat diet have been found to have a shorter lifespan than their wild-type counterpart, and mice without both receptors have an even shorter survival. AdipoR receptors help in the combustion of fatty acids, increase antioxidant capacity and reduce the concentration of pro-inflammatory cytokines (Okada-Iwabu et al. 2013). Due to all these findings, much work is currently being done on the design of small molecule agonists or activating antibodies against AdipoRs, mainly for the treatment of diseases, but additionally these drugs could also cause a greater lifespan and healthspan (Yamauchi and Kadowaki 2013; Okada-Iwabu et al. 2015, 2018).

### Other GPCRs

#### Studied in *Drosophila*

Silencing isoform B of the metabotropic glutamate receptor (mGluR) in *Drosophila* reduced both median and maximum lifespan, possibly by not being able to inhibit ionotropic glutamate receptor-mediated calcium increases that lead to increased cellular excitability during periods when inhibitory modulation is required such as during the sleep. Thus, in young flies there is an increase in sleep during the day and a decrease in sleep at night, while in older flies it exacerbates age-related sleep loss (Ly and Naidoo 2019). Another mechanism that could be involved, although it has not been tested experimentally or in this study model, is through the regulation of growth hormone (GH) secretion (Tena-Sempere et al. 2000).

Another GPCR with effects on *Drosophila* lifespan is CapaR, the receptor for neuropeptides CAP-1 and CAP-2 that are homologous to mammalian neuromedin U and are known to influence ion and water balance by regulating the activity of the renal tubules of Malpighi (Sajadi et al. 2020). Similar to what occurs with AGTR2, the increased activity of CapaR increases the excretion of fluids and wastes, inhibits the release of glucagon-like adipokinetic hormone from the cardiac body, which restricts the mobilization of energy from adipose tissue and prevents the harmful hyperglycemia. In contrast, the loss of CapaR causes intestinal hypomotility and nutrient malabsorption, gradually depleting internal nutrient stores and reducing the life span of the insect (Koyama et al. 2021).

The decrease in the expression of the tachykinin-like peptides receptor 99D (TkR99D) in IPCs made *Drosophila* more sensitive to starvation, reducing its lifespan under these conditions. TkR99D knockdown-flies with 24 h of starvation increased Dilp2 levels while those of Dilp3 decreased, without significant changes in Dilp5. They also showed a faster reduction in trehalose levels but no effect on lipid levels (Birse et al. 2011). On the contrary, low levels of TkR99D in the main cells of the renal tubules of *Drosophila* increased their survival against desiccant, nutritional and oxidative stress, while its overexpression had opposite effects. This could occur since TkR99D negatively controls the release of Dilp5 in these cells and the diuretic activity of the tubules (Söderberg et al. 2011).

Bride of sevenless (BOSS) is a protein necessary for the development of the R7 photoreceptor neuron in the *Drosophila* compound eye and in the maintenance of energy homeostasis. Flies deficient in BOSS have a shorter life expectancy, which is related to an increase in reactive oxygen species (ROS), a decrease in the concentration of antioxidant enzymes such as SOD2, an increase in mitochondrial mass and a greater amount of protein adducts such as advanced lipoxidation end products and glycation end products (Kohyama-Koganeya et al. 2017).

#### Studied in *Caenorhabditis*

Loss-of-function mutations in the natriuretic peptide receptor 1 (NPR1) were shown to extend the life of *Caenorhabditis*, and this effect was enhanced by a deletion mutation in the atypical soluble guanylate cyclase GCY35 oxygen sensor possibly through a mechanism involving neuropeptide signal transduction and ROS. This was observed mainly when the receptors located in the oxygen-sensitive neurons AQR, PQR, URX, BAG and the interneuron RIA were affected. In addition, there is an important role for guanylate cyclase GCY-33, as well as insulin signaling pathways that are activated by the transcription factors HIF-1 and DAF-16 (Abergel et al. 2017). Meanwhile, the elimination of NPR-22 prevented a greater survival of worms that overexpress the pharyngeal neuropeptides LURY-1, mainly when this was done in MC neurons (Ohno et al. 2017).

Loss of the G-protein-linked acetylcholine receptor 3 (GAR-3), which acts as an extrasynaptic detector of acetylcholine levels and is important in responding to nutrient conditions, was shown to prolong the lifespan of *Caenorhabditis* worms. It is thought that the abundance of food induces an increase in acetylcholine, which is detected by GAR-3 in the pharyngeal muscles, so when this receptor is lost, the frequency of contraction of the pharyngeal muscle decreases and causes a phenomenon similar to that of occurs with dietary restriction (Lucanic et al. 2016). This is also similar to what happens with DOP-3 (See section “Dopamine receptors”) and the vesicular acetylcholine transporter (VAChT) in *Drosophila* (Showell et al. 2020).

Meanwhile, the transmembrane receptor 2 (STR-2) expressed in the amphidic sensory neurons AWC and ASI was shown to control lipid droplet homeostasis and lipid metabolism in the gut of these worms by regulating the expression of delta-9 desaturases, FAT-5, FAT-6, and FAT-7, the diacylglycerol acyltransferase DGAT-2, the lipase LIPL-3, and the acyl-CoA synthetase ACS-2. Thus, STR-2 was shown to be required when *Caenorhabditis* grows at temperatures above 20 °C by maintaining the levels of stored fats, mainly monounsaturated fatty acids (MUFAs), that are required for longevity (Dixit et al. 2020).

*Caenorhabditis* worms that do not express octopamine G protein-couple receptor 1 (OCTR-1) in ASH chemosensory neurons live much longer at temperatures above 25 °C than wild-type animals due to down-regulation of immune defense genes such as LYS-5, ACDH -1, LYS-4, and SRX-128 that possibly prevent chronic inflammation with aging or other harmful immune phenomena (Wibisono et al. 2021). Although it remains to be verified, SER-3 could also be involved (See section “Serotonin receptors”).

Also acting through the regulation of the immune system, the absence of *Caenorhabditis* SRBC-48 produced a shorter lifespan of the worms after uncontrolled activation of immune genes, particularly those regulated by the transcription factor DAF-16 of the FOXO family, which favors the *Pseudomonas aeruginosa* infection and consequently anterior dendritic degeneration (Kaur and Aballay 2020).

Lastly, the expression of DAF-37 in *Caenorhabditis* ASK neurons was shown to be important in causing Sir2.1-dependent increases in lifespan and stress resistance triggered by some ascarosides, components of a population density signal (Ludewig et al. 2013).

#### Studied in rodents

In the hypothalamus of mice, specifically in the dorsomedial and lateral nuclei, SIRT1 and NKX2-1 cooperate to upregulate hypocretin receptor type 2 (HCRTR2) expression with consequent neural activation. Enhanced neural activity stimulates the sympathetic nervous system and maintains skeletal muscle mitochondrial morphology and function, physical activity, body temperature, and oxygen consumption during aging. This neural activation of the SIRT1/NKX2-1/HCRTR2 circuit is also essential to preserve the quality of sleep during aging. Thus, all of these functional benefits contribute to the maintenance of youthful physiology and promote longevity (Satoh et al. 2013).

Bile acids prolong the longevity of yeast, flies, worms and mice by activating the nuclear farnesoid receptor X (FXF) and its consequent cytoprotective and anticancer properties (Krøll 2012). The joint activation of the G protein-coupled bile acid receptor 1 (GPBAR1) and FXR in old mice can act as caloric restriction mimetics and reverse some age-related changes, such as increased urinary albumin excretion, decreased mitochondrial function and biogenesis, and kidney inflammation. Reinforcing the role of both receptors in longevity, it was also observed that the expression of GPBAR1 and FXR is higher in the kidneys of long-lived Ames dwarf mice than in short-lived mice (Wang et al. 2017b).

G protein-coupled estrogen receptor 1 (GPER) mediates many of the non-genomic effects of estrogens and therefore plays an important role in various aspects of aging, including vascular and neurological aging, memory, and synaptic plasticity. This occurs through its intrinsic signaling, by interacting with ER, IGF-1R and EGFR and, to some extent, the regulation of gene expression (Yang et al. 2021). GPER activation and overexpression in SH-SY5Y neuroblastoma cells was shown to reduce apoptosis induced by the neurotoxic 1-methyl-4 phenylpyridinium (MPP+) (Cheng et al. 2017). Furthermore, it improves memory in old and middle-aged mice by stimulating BDNF/TrkB signaling that causes ARC synthesis and internalization and degradation of the glutamate receptor GluA1, particularly in the CA3 region of the hippocampus (Briz et al. 2015; Xu et al. 2018). However, the activation of GPER also causes the reduction of PTEN levels and the activation of AKT and mTOR, which contrasts with the events described that cause an increase in longevity, but an experimental confirmation is necessary to reach a conclusion (Briz et al. 2015).

Deletion of the formyl peptide receptor-related sequence 8 (FPR-rs8) in mice, a receptor for chemoattractants of phagocytes such as N-formylpeptides derived from bacteria and mitochondria, have reduced longevity (Tiffany et al. 2011).

#### Studied in humans

The SNP rs6166 in the follicle-stimulating hormone (FSH) receptor (FSHR), where asparagine 680 is changed by a serine, was linked in women with a higher probability of living more than 90 years, possibly due to lower fertility and according to the longevity–fertility trade-off hypothesis (Corbo et al. 2013). Coupled with this, the reduction in FSH levels causes the activation of BAT, the browning of the white adipose tissue (WAT) and a higher energy expenditure that are associated with important extensions of health and longevity (Liu et al. 2017; Bartke 2017).

It has been mentioned that the SNP rs333 in which a 32 bp deletion occurs in exon 4 of the CCR5 gene and generates a non-functional variant called CCR5Δ32 was overrepresented in centenarians (Balistreri et al. 2008). However, other studies did not find statistical evidence for an effect of CCR5∆32 on lifespan (Maier et al. 2020).

## Conclusions

Due to their great pharmacological manageability and their important role in the pathophysiology of living beings, GPCRs could be important targets in trying to prolong our lifespan and healthspan. Although most of the findings on this topic have been made mainly indirectly, the analysis of its role in lifespan could be widely analyzed and lead to notable improvements in our quality of life. We could use both agonists for those GPCRs with beneficial effects and antagonists for those with harmful effects, as well as midpoints with partial and inverse agonists (Supplementary Table 1). However, before moving forward, we would first have to decide whether it is feasible to use this type of therapy at the cost of limiting the perception of some of our senses. It is necessary to study in depth the effects of enhancing or inhibiting the signaling of each GPCR for a long time, the appearance of drug resistance phenomena and possible side effects. Another important aspect is to understand the mechanisms associated with the increase in lifespan. Through this text it can be observed how GPCRs influence lifespan through different mechanisms, highlighting those that mimic dietary restriction, those related to insulin signaling and the AMPK and TOR pathways, and those that alter oxidative homeostasis and severe and/or chronic inflammation, among others (Table 1). However, even more research is needed in this area and future studies should perform high-throughput analysis considering genomic, transcriptomic, epigenomic and proteomic data, among many others, trying to integrate them all. In this sense, the use of artificial intelligence (AI) could be of great help, but it should be implemented gradually so that we do it correctly and with critical thinking. Gene editing systems should be exploited to create models more similar to the human context in order to confirm and validate the magnitude of the impact of each GPCR. Finally, the development of new drugs directed against GPCRs, more specific, with fewer side effects and more accessible is a desirable prospect.

## Supplementary Information

Below is the link to the electronic supplementary material.

Supplementary file1 (DOCX 54 kb)
