# Supplementary material for: G protein-coupled receptors that influence lifespan of human and animal models
Source: Biogerontology. 2021 Dec 3;23(1):1–19. doi: 10.1007/s10522-021-09945-8 (PMC8888397; doi:10.1007/s10522-021-09945-8)
Supplement: Supplementary file 1 — Supplementary file1 (DOCX 54 kb) [file 10522_2021_9945_MOESM1_ESM.docx]

**Supplementary Table 1. Drugs that act on GPCRs with effects on lifespan.**

| **GPCR** | **Drug Name** | **Disease in which the drug is used** | **Drug Type** | **Status** | **Mechanism Of Action** |
| --- | --- | --- | --- | --- | --- |
| HTR2A | Sertindole | Schizophrenia | Small Molecule | Withdrawn | Antagonist |
|  | Acp-104 | Schizophrenia | Small Molecule | In Trial | Inverse Agonist |
|  | Am-831 | Antipsychotic ; Schizophrenia | Small Molecule | In Trial | Antagonist |
|  | Apd125 | Insomnia | Small Molecule | In Trial | Agonist |
|  | Apd125 | Dementia With Lewy Bodies | Small Molecule | In Trial | Agonist |
|  | Apd791 | Antithrombotic | Small Molecule | In Trial | Agonist |
|  | Ati-9242 | Schizophrenia | Small Molecule | In Trial | Not Available |
|  | Bifeprunox | Antipsychotic ; Antiparkinson | Small Molecule | In Trial | Agonist |
|  | Bl-1020 | Schizophrenia | Small Molecule | In Trial | Agonist |
|  | Bupropion/Trazodone | Anti-Depressant; For Hypoactive Sexual Desire Disorder And Female Sexual Dysfunction | Small Molecule | In Trial | Antagonist |
|  | Bvt.28949 | Antiglaucomic | Small Molecule | In Trial | Antagonist |
|  | Cis-Uca Eye Drops | For Ocular Surface Inflammation | Small Molecule | In Trial | Agonist |
|  | Cyclobenzaprine | Fibromyalgia, Ptsd | Small Molecule | In Trial | Antagonist |
|  | Eplivanserine | Insomnia | Small Molecule | In Trial | Inverse Agonist |
|  | Esmirtazapine | Insomnia; Menopausal Symptoms | Small Molecule | In Trial | Antagonist |
|  | Iferanserin | Antihemorrhoidal | Small Molecule | In Trial | Antagonist |
|  | Iti-007 | Schizophrenia | Small Molecule | In Trial | Antagonist |
|  | Min-001, Cyr-101 | Schizophrenia | Small Molecule | In Trial | Agonist |
|  | Min-117 | Anti-Depressant | Small Molecule | In Trial | Antagonist |
|  | Nelotanserin, Apd-125 | Dementia; Parkinson's Disease | Small Molecule | In Trial | Inverse Agonist |
|  | Nemifitide | Anti-Depressant | Peptide | In Trial | Antagonist |
|  | Sl 650472 | Intermittent Claudication | Small Molecule | In Trial | Antagonist |
|  | Syn120 | Cognitive Disorders | Small Molecule | In Trial | Antagonist |
|  | Temanogrel | Acute Coronary Syndrome | Small Molecule | In Trial | Inverse Agonist |
|  | Terguride | Diffuse Cutaneous Systemic Sclerosis, Antihypertensive | Small Molecule | In Trial | Antagonist |
|  | Volinanserin | Hypnotics And Sedatives | Small Molecule | In Trial | Antagonist |
|  | Ykp-1358 | Schizophrenia | Unknown | In Trial | Antagonist |
|  | Renzapride | Irritable Bowel Syndrome | Small Molecule | In Trial | Antagonist |
|  | Vabicaserin | Schizophrenia | Small Molecule | In Trial | Agonist |
|  | Acepromazine, Aceprometazine | Antipsychotic | Small Molecule | Approved | Antagonist |
|  | [Amitriptyline](file:////drugs/nhs/amitriptyline%20) | Analgesics | Small Molecule | Approved | Antagonist |
|  | [Aripiprazole](file:////drugs/nhs/aripiprazole%20) | Schizophrenia | Small Molecule | Approved | Partial Agonist |
|  | [Aripiprazole](file:////drugs/nhs/aripiprazole%20) | Psychosis | Small Molecule | Approved | Partial Agonist |
|  | [Asenapine](file:////drugs/nhs/asenapine%20) | Schizophrenia | Small Molecule | Approved | Antagonist |
|  | Brexpiprazole | Anti-Depressant | Small Molecule | Approved | Partial Agonist |
|  | Butriptyline | Anti-Depressant | Small Molecule | Approved | Antagonist |
|  | [Chlorpromazine](file:////drugs/nhs/chlorpromazine%20) | Psychosis | Small Molecule | Approved | Antagonist |
|  | [Chlorprothixene](file:////drugs/nhs/chlorprothixene%20) | Schizophrenia | Small Molecule | Approved | Antagonist |
|  | Cinitapride | Gastrointestinal Disorders | Small Molecule | Approved | Antagonist |
|  | Cyclobenzaprine | Muscle Relaxants | Small Molecule | Approved | Antagonist |
|  | [Clozapine](file:////drugs/nhs/clozapine%20) | Schizophrenia | Small Molecule | Approved | Antagonist |
|  | Cyclobenzaprine | Anti-Depressant | Small Molecule | Approved | Antagonist |
|  | [Cyproheptadine](file:////drugs/nhs/cyproheptadine%20) | Anti-Allergic ; Appetite Stimulant | Small Molecule | Approved | Antagonist |
|  | [Doxepin](file:////drugs/nhs/doxepin%20) | Hypnotics And Sedatives | Small Molecule | Approved | Antagonist |
|  | Droperidol | Psychosis | Small Molecule | Approved | Antagonist |
|  | [Flupentixol](file:////drugs/nhs/flupentixol%20) | Antipsychotic | Small Molecule | Approved | Antagonist |
|  | Iloperidone | Antipsychotic ; Atypical | Small Molecule | Approved | Antagonist |
|  | [Loxapine](file:////drugs/nhs/loxapine%20) | Schizophrenia | Small Molecule | Approved | Antagonist |
|  | [Lurasidone](file:////drugs/nhs/lurasidone%20) | Schizophrenia | Small Molecule | Approved | Antagonist |
|  | Mesoridazine | Schizophrenia | Small Molecule | Approved | Antagonist |
|  | [Methysergide](file:////drugs/nhs/methysergide%20) | Anti-Migraine S; Vasoconstrictor | Small Molecule | Approved | Antagonist |
|  | [Mianserin](file:////drugs/nhs/mianserin%20) | Anti-Depressant | Small Molecule | Approved | Antagonist |
|  | Minaprine | Anti-Depressant | Small Molecule | Approved | Antagonist |
|  | [Mirtazapine](file:////drugs/nhs/mirtazapine%20) | Anti-Depressant | Small Molecule | Approved | Antagonist |
|  | [Nefazodone](file:////drugs/nhs/nefazodone%20) | Anti-Depressant | Small Molecule | Approved | Antagonist |
|  | [Olanzapine](file:////drugs/nhs/olanzapine%20) | Anti-Depressant | Small Molecule | Approved | Antagonist |
|  | [Olanzapine](file:////drugs/nhs/olanzapine%20) | Schizophrenia | Small Molecule | Approved | Antagonist |
|  | [Paliperidone](file:////drugs/nhs/paliperidone%20) | Schizophrenia | Small Molecule | Approved | Antagonist |
|  | Pimavanserin | Antiparkinson | Small Molecule | Approved | Inverse Agonist |
|  | [Pipotiazine](file:////drugs/nhs/pipotiazine%20) | Antipsychotic | Small Molecule | Approved | Antagonist |
|  | [Promazine](file:////drugs/nhs/promazine%20) | Antiemetics; Antipsychotic | Small Molecule | Approved | Antagonist |
|  | Propiomazine | Hypnotics And Sedatives | Small Molecule | Approved | Antagonist |
|  | [Quetiapine](file:////drugs/nhs/quetiapine%20) | Antipsychotic ; Anti-Depressant | Small Molecule | Approved | Antagonist |
|  | [Risperidone](file:////drugs/nhs/risperidone%20) | Schizophrenia | Small Molecule | Approved | Antagonist |
|  | Thiethylperazine | Antiemetics | Small Molecule | Approved | Antagonist |
|  | Thioproperazine | Antipsychotic | Small Molecule | Approved | Antagonist |
|  | [Thioridazine](file:////drugs/nhs/thioridazine%20) | Schizophrenia | Small Molecule | Approved | Antagonist |
|  | Thiothixene | Psychosis | Small Molecule | Approved | Antagonist |
|  | [Trazodone](file:////drugs/nhs/trazodone%20) | Anti-Depressant | Small Molecule | Approved | Partial Agonist |
|  | [Trimipramine](file:////drugs/nhs/trimipramine%20) | Unipolar Depression | Small Molecule | Approved | Antagonist |
|  | [Ziprasidone](file:////drugs/nhs/ziprasidone%20) | Schizophrenia | Small Molecule | Approved | Antagonist |
|  | Flibanserin | Sexual Dysfunction | Small Molecule | Approved | Antagonist |
|  | [Haloperidol](file:////drugs/nhs/haloperidol%20) | Conduct Disorder | Small Molecule | Approved | Antagonist |
|  | [Haloperidol](file:////drugs/nhs/haloperidol%20) | Psychosis | Small Molecule | Approved | Antagonist |
|  | Molindone | Schizophrenia | Small Molecule | Approved | Antagonist |
|  | [Trifluoperazine](file:////drugs/nhs/trifluoperazine%20) | Psychosis | Small Molecule | Approved | Antagonist |
| DRD2 | Sertindole | Schizophrenia | Withdrawn | Small Molecule | Antagonist |
|  | Acp-104 | Schizophrenia | In Trial | Small Molecule | Partial Agonist |
|  | Amisulpride | Schizophrenia | In Trial | Small Molecule | Antagonist |
|  | Am-831 | Antipsychotic ; Schizophrenia | In Trial | Small Molecule | Antagonist |
|  | Amisulpride | Antiemetics | In Trial | Small Molecule | Antagonist |
|  | Aplindore | Antiparkinson ; Restlegs Legs Syndrome | In Trial | Small Molecule | Partial Agonist |
|  | [Apomorphine](file:////drugs/nhs/apomorphine%20) | Erectile Dysfunction | In Trial | Small Molecule | Agonist |
|  | [Apomorphine](file:////drugs/nhs/apomorphine%20) | Sexual Dysfunction In Women; Erectile Dysfunction; Antiparkinson | In Trial | Small Molecule | Agonist |
|  | [Apomorphine](file:////drugs/nhs/apomorphine%20) | Sexual Dysfunction In Women; Erectile Dysfunction; Antiparkinson | In Trial | Small Molecule | Agonist |
|  | [Apomorphine](file:////drugs/nhs/apomorphine%20) | Antiparkinson | In Trial | Small Molecule | Agonist |
|  | Ati-9242 | Schizophrenia | In Trial | Small Molecule | Not Available |
|  | Bifeprunox | Antipsychotic ; Antiparkinson | In Trial | Small Molecule | Partial Agonist |
|  | Bim23a760 | Antineoplastic ; Treatment For Acromegaly | In Trial | Small Molecule | Agonist |
|  | Bl-1020 | Schizophrenia | In Trial | Small Molecule | Antagonist |
|  | Domperidone | Antiemetics | In Trial | Small Molecule | Antagonist |
|  | Etilevodopa | Antiparkinson | In Trial | Small Molecule | Agonist |
|  | Flupenthixol | Schizophrenia | In Trial | Small Molecule | Antagonist |
|  | Iti-007 | Schizophrenia | In Trial | Small Molecule | Antagonist |
|  | Itopride | Motilitant | In Trial | Small Molecule | Antagonist |
|  | Jnj-37822681 | Schizophrenia | In Trial | Small Molecule | Antagonist |
|  | [Levodopa](file:////drugs/nhs/levodopa%20) | Antiparkinson | In Trial | Small Molecule | Agonist |
|  | [Levodopa](file:////drugs/nhs/levodopa%20) | Antiparkinson | In Trial | Small Molecule | Agonist |
|  | Lu 02-750 | Antiparkinson | In Trial | Unknown | Unknown |
|  | [Metoclopramide](file:////drugs/nhs/metoclopramide%20) | Diabetic Gastroparesis | In Trial | Small Molecule | Antagonist |
|  | Lu Ae04621 | Antiparkinson | In Trial | Unknown | Agonist |
|  | [Metoclopramide](file:////drugs/nhs/metoclopramide%20) | Anti-Migraine | In Trial | Small Molecule | Antagonist |
|  | Molindone | Adhd | In Trial | Small Molecule | Antagonist |
|  | Ordopidine | Antiparkinson | In Trial | Small Molecule | Not Available |
|  | [Pramipexole](file:////drugs/nhs/pramipexole%20) | Antiparkinson | In Trial | Small Molecule | Agonist |
|  | Pardoprunox | Antiparkinson | In Trial | Small Molecule | Agonist |
|  | Pridopidine | Huntington's Disease | In Trial | Small Molecule | Antagonist |
|  | [Prochlorperazine](file:////drugs/nhs/prochlorperazine%20) | Anti-Migraine | In Trial | Small Molecule | Antagonist |
|  | [Ropinirole](file:////drugs/nhs/ropinirole%20) | Restless Legs Syndrome | In Trial | Small Molecule | Agonist |
|  | Sulpiride | Antidepressive ; Second-Generation; Antipsychotic | In Trial | Small Molecule | Antagonist |
|  | Xp21279 | Antiparkinson | In Trial | Small Molecule | Agonist |
|  | Ykp-1358 | Schizophrenia | In Trial | Unknown | Antagonist |
|  | Dexpramipexole | Amyotrophic Lateral Sclerosis (Als) | In Trial | Small Molecule | Agonist |
|  | Acepromazine, Aceprometazine | Antipsychotic | Approved | Small Molecule | Antagonist |
|  | Acetophenazine | Schizophrenia | Approved | Small Molecule | Antagonist |
|  | Amantadine Hcl | Dyskinesias | Approved | Small Molecule | Antagonist |
|  | [Amoxapine](file:////drugs/nhs/amoxapine%20) | Unipolar Depression | Approved | Small Molecule | Antagonist |
|  | [Apomorphine](file:////drugs/nhs/apomorphine%20) | Anti-Parkisonian | Approved | Small Molecule | Agonist |
|  | [Aripiprazole](file:////drugs/nhs/aripiprazole%20) | Schizophrenia | Approved | Small Molecule | Partial Agonist |
|  | [Aripiprazole](file:////drugs/nhs/aripiprazole%20) | Psychosis | Approved | Small Molecule | Partial Agonist |
|  | [Asenapine](file:////drugs/nhs/asenapine%20) | Schizophrenia | Approved | Small Molecule | Antagonist |
|  | [Benperidol](file:////drugs/nhs/benperidol%20) | Antipsychotic | Approved | Small Molecule | Antagonist |
|  | [Bromocriptine](file:////drugs/nhs/bromocriptine%20) | Antidiabetic | Approved | Small Molecule | Agonist |
|  | Brexpiprazole | Anti-Depressant | Approved | Small Molecule | Partial Agonist |
|  | Bromopride | Antiemetics | Approved | Small Molecule | Antagonist |
|  | [Buspirone](file:////drugs/nhs/buspirone%20) | Anti-Anxiety | Approved | Small Molecule | Partial Agonist |
|  | [Cabergoline](file:////drugs/nhs/cabergoline%20) | Antiparkinson | Approved | Small Molecule | Agonist |
|  | Cariprazine | Schizophrenia | Approved | Small Molecule | Partial Agonist |
|  | Carphenazine | Schizophrenia | Approved | Small Molecule | Antagonist |
|  | [Chlorpromazine](file:////drugs/nhs/chlorpromazine%20) | Psychosis | Approved | Small Molecule | Antagonist |
|  | [Chlorprothixene](file:////drugs/nhs/chlorprothixene%20) | Schizophrenia | Approved | Small Molecule | Antagonist |
|  | [Clozapine](file:////drugs/nhs/clozapine%20) | Schizophrenia | Approved | Small Molecule | Antagonist |
|  | Droperidol | Adjuvants; Anesthesia | Approved | Small Molecule | Antagonist |
|  | [Flupentixol](file:////drugs/nhs/flupentixol%20) | Antipsychotic | Approved | Small Molecule | Antagonist |
|  | [Fluphenazine](file:////drugs/nhs/fluphenazine%20) | Schizophrenia | Approved | Small Molecule | Antagonist |
|  | [Fluphenazine Decanoate](file:////drugs/nhs/fluphenazine%20decanoate%20) | Psychosis | Approved | Small Molecule | Antagonist |
|  | Fluphenazine Enanthate | Psychosis | Approved | Small Molecule | Antagonist |
|  | Fluspirilene | Schizophrenia | Approved | Small Molecule | Antagonist |
|  | [Haloperidol](file:////drugs/nhs/haloperidol%20) | Psychosis | Approved | Small Molecule | Antagonist |
|  | [Haloperidol](file:////drugs/nhs/haloperidol%20) | Schizophrenia | Approved | Small Molecule | Antagonist |
|  | Iloperidone | Antipsychotic ; Atypical | Approved | Small Molecule | Antagonist |
|  | [Levodopa](file:////drugs/nhs/levodopa%20) | Antiparkinson | Approved | Small Molecule | Agonist |
|  | [Levodopa](file:////drugs/nhs/levodopa%20) | Antiparkinson | Approved | Small Molecule | Agonist |
|  | [Levomepromazine](file:////drugs/nhs/levomepromazine%20) | Psychosis | Approved | Small Molecule | Antagonist |
|  | Lisuride | Antiparkinson | Approved | Small Molecule | Agonist |
|  | [Loxapine](file:////drugs/nhs/loxapine%20) | Schizophrenia | Approved | Small Molecule | Antagonist |
|  | [Lurasidone](file:////drugs/nhs/lurasidone%20) | Schizophrenia | Approved | Small Molecule | Antagonist |
|  | Melevodopa | Antiparkinson | Approved | Small Molecule | Agonist |
|  | Mesoridazine | Schizophrenia | Approved | Small Molecule | Antagonist |
|  | [Metoclopramide](file:////drugs/nhs/metoclopramide%20) | Motilitant; Gastroesophageal Reflux Disease | Approved | Small Molecule | Antagonist |
|  | [Metoclopramide](file:////drugs/nhs/metoclopramide%20) | Antiemetics | Approved | Small Molecule | Antagonist |
|  | Molindone | Schizophrenia | Approved | Small Molecule | Antagonist |
|  | [Olanzapine](file:////drugs/nhs/olanzapine%20) | Anti-Depressant | Approved | Small Molecule | Antagonist |
|  | [Olanzapine](file:////drugs/nhs/olanzapine%20) | Schizophrenia | Approved | Small Molecule | Antagonist |
|  | [Paliperidone](file:////drugs/nhs/paliperidone%20) | Schizophrenia | Approved | Small Molecule | Antagonist |
|  | [Pergolide](file:////drugs/nhs/pergolide%20) | Antiparkinson | Approved | Small Molecule | Agonist |
|  | [Perphenazine](file:////drugs/nhs/perphenazine%20) | Schizophrenia | Approved | Small Molecule | Antagonist |
|  | [Pimozide](file:////drugs/nhs/pimozide%20) | Antidyskinetics; Antipsychotic | Approved | Small Molecule | Antagonist |
|  | [Pipotiazine](file:////drugs/nhs/pipotiazine%20) | Antipsychotic | Approved | Small Molecule | Antagonist |
|  | [Pramipexole](file:////drugs/nhs/pramipexole%20) | Antiparkinson | Approved | Small Molecule | Agonist |
|  | [Prochlorperazine](file:////drugs/nhs/prochlorperazine%20) | Antiemetics | Approved | Small Molecule | Antagonist |
|  | [Promazine](file:////drugs/nhs/promazine%20) | Antiemetics; Antipsychotic | Approved | Small Molecule | Antagonist |
|  | Propiomazine | Hypnotics And Sedatives | Approved | Small Molecule | Antagonist |
|  | [Quetiapine](file:////drugs/nhs/quetiapine%20) | Antipsychotic ; Anti-Depressant | Approved | Small Molecule | Antagonist |
|  | Remoxipride | Schizophrenia | Approved | Small Molecule | Antagonist |
|  | [Ropinirole](file:////drugs/nhs/ropinirole%20) | Antiparkinson | Approved | Small Molecule | Agonist |
|  | [Risperidone](file:////drugs/nhs/risperidone%20) | Schizophrenia | Approved | Small Molecule | Antagonist |
|  | [Rotigotine](file:////drugs/nhs/rotigotine%20) | Antiparkinson | Approved | Small Molecule | Agonist |
|  | Thiethylperazine | Antiemetics | Approved | Small Molecule | Antagonist |
|  | Thioproperazine | Antipsychotic | Approved | Small Molecule | Antagonist |
|  | [Thioridazine](file:////drugs/nhs/thioridazine%20) | Schizophrenia | Approved | Small Molecule | Antagonist |
|  | Thiothixene | Psychosis | Approved | Small Molecule | Antagonist |
|  | [Trifluoperazine](file:////drugs/nhs/trifluoperazine%20) | Antiemetics; Antipsychotic | Approved | Small Molecule | Antagonist |
|  | Triflupromazine | Antiemetics; Antipsychotic | Approved | Small Molecule | Antagonist |
|  | [Trimipramine](file:////drugs/nhs/trimipramine%20) | Unipolar Depression | Approved | Small Molecule | Antagonist |
|  | [Ziprasidone](file:////drugs/nhs/ziprasidone%20) | Schizophrenia | Approved | Small Molecule | Antagonist |
|  | [Zuclopenthixol](file:////drugs/nhs/zuclopenthixol%20) | Schizophrenia | Approved | Small Molecule | Antagonist |
|  | [Ergoloid](file:////drugs/nhs/ergoloid%20) | Cardiovascular Disease | Approved | Small Molecule | Agonist |
|  | Minaprine | Anti-Depressant | Approved | Small Molecule | Antagonist |
| AGTR2 | Ema401 | Peripheral Neuropathic Pain; Post Herpetic Neuralgia | Small Molecule | In Trial | Antagonist |
|  | Ljpc-501 | Hepatorenal Syndrome-Catecholamine-Resistant Hypotension (CRH) | Peptide | In Trial | Agonist |
|  | MOR107 | Diabetic Kidney Disease | Peptide | In Trial | Agonist |
|  | Ps433540 | Focal Segmental Glomerulosclerosis, Antihypertensive | Small Molecule | In Trial | Antagonist |
| GPBAR1 | Int-767 | Treatment Of Liver Fibrosis | Small Molecule | In Trial | Agonist |
| ADRB2 | Abediterol | Anti-Asthmatic | Small Molecule | In Trial | Agonist |
|  | Asm-024 | Anti-Asthmatic ; COPD | Small Molecule | In Trial | Agonist |
|  | Azd3199 | Chronic Obstructive Pulmonary Disease (COPD) | Small Molecule | In Trial | Agonist |
|  | AZD8871 | Anti-Asthmatic | Small Molecule | In Trial | Agonist |
|  | Batefenterol | Chronic Obstructive Pulmonary Disease (COPD) | Small Molecule | In Trial | Agonist |
|  | Bedoradrine | Anti-Asthmatic | Small Molecule | In Trial | Agonist |
|  | Bucindolol | Heart Failure | Small Molecule | In Trial | Antagonist |
|  | Carmoterol | Chronic Obstructive Pulmonary Disease (COPD) | Small Molecule | In Trial | Agonist |
|  | [Formoterol](file:////drugs/nhs/formoterol%20) | Chronic Obstructive Pulmonary Disease (COPD) | Small Molecule | In Trial | Agonist |
|  | [Formoterol](file:////drugs/nhs/formoterol%20) | Bronchodilator | Small Molecule | In Trial | Agonist |
|  | [Formoterol](file:////drugs/nhs/formoterol%20) | Appetite Stimulant | Small Molecule | In Trial | Agonist |
|  | Gsk-961081 | Bronchodilator | Small Molecule | In Trial | Agonist |
|  | Inv102, Nadolol | For Inflammatory Lung Disease | Small Molecule | In Trial | Inverse Agonist |
|  | Las-100977 | Bronchodilator | Small Molecule | In Trial | Agonist |
|  | Milveterol | Bronchodilator | Small Molecule | In Trial | Agonist |
|  | Mn-221 | Anti-Asthmatic | Unknown | In Trial | Agonist |
|  | Ot-730 | Antiglaucomic | Small Molecule | In Trial | Antagonist |
|  | Pf-00610355 | Bronchodilator | Small Molecule | In Trial | Agonist |
|  | Procaterol | Bronchodilator | Small Molecule | In Trial | Agonist |
|  | Pw2101 | Antihypertensive | Small Molecule | In Trial | Antagonist |
|  | Syl040012 | Antiglaucomic | Sirna | In Trial | Antagonist |
|  | [Vilanterol](file:////drugs/nhs/vilanterol%20) | Chronic Obstructive Pulmonary Disease (COPD) | Small Molecule | In Trial | Agonist |
|  | Cicletanine | Antihypertensive | Small Molecule | In Trial | Not Available |
|  | Albuterol | Chronic Obstructive Pulmonary Disease (COPD) | Small Molecule | Approved | Agonist |
|  | Alprenolol | Anti-Arrhythmia ; Antihypertensive | Small Molecule | Approved | Antagonist |
|  | Arformoterol | Bronchodilator | Small Molecule | Approved | Agonist |
|  | Bitolterol | Obstructive Lung Disease | Small Molecule | Approved | Agonist |
|  | [Carteolol](file:////drugs/nhs/carteolol%20) | Cardiovascular Disease | Small Molecule | Approved | Antagonist |
|  | [Celiprolol](file:////drugs/nhs/celiprolol%20) | Antihypertensive | Small Molecule | Approved | Agonist |
|  | Clenbuterol | Bronchodilator | Small Molecule | Approved | Agonist |
|  | [Dipivefrin](file:////drugs/nhs/dipivefrin%20) | Antiglaucomic | Small Molecule | Approved | Agonist |
|  | Droxidopa | Orthostatic Hypotension | Small Molecule | Approved | Agonist |
|  | Epinephrine, Adrenaline | Anesthetics | Small Molecule | Approved | Agonist |
|  | [Fenoterol](file:////drugs/nhs/fenoterol%20) | Bronchodilator ; Tocolytic | Small Molecule | Approved | Agonist |
|  | [Formoterol](file:////drugs/nhs/formoterol%20) | Anti-Asthmatic | Small Molecule | Approved | Agonist |
|  | [Formoterol](file:////drugs/nhs/formoterol%20) | Anti-Asthmatic | Small Molecule | Approved | Agonist |
|  | [Formoterol](file:////drugs/nhs/formoterol%20) | Chronic Obstructive Pulmonary Disease (COPD) | Small Molecule | Approved | Agonist |
|  | [Formoterol](file:////drugs/nhs/formoterol%20) | Chronic Obstructive Pulmonary Disease (COPD) | Small Molecule | Approved | Agonist |
|  | [Formoterol](file:////drugs/nhs/formoterol%20) | Bronchodilator | Small Molecule | Approved | Agonist |
|  | [Formoterol](file:////drugs/nhs/formoterol%20) | Anti-Asthmatic ; COPD | Small Molecule | Approved | Agonist |
|  | [Glycopyrronium](file:////drugs/nhs/glycopyrronium%20) | Chronic Obstructive Pulmonary Disease (COPD) | Small Molecule | Approved | Antagonist |
|  | [Indacaterol](file:////drugs/nhs/indacaterol%20) | Bronchodilator | Small Molecule | Approved | Agonist |
|  | Isoproterenol | Cardiovascular ; Anti-Asthmatic | Small Molecule | Approved | Agonist |
|  | Isoproterenol | Bronchodilator ; Cardiotonic | Small Molecule | Approved | Agonist |
|  | [Labetalol](file:////drugs/nhs/labetalol%20) | Antihypertensive | Small Molecule | Approved | Antagonist |
|  | [Levobunolol](file:////drugs/nhs/levobunolol%20) | Antiglaucomic | Small Molecule | Approved | Antagonist |
|  | Levosalbutamol | Anti-Asthmatic | Small Molecule | Approved | Agonist |
|  | Mephentermine | Cardiovascular Disease | Small Molecule | Approved | Agonist |
|  | Metaproterenol, Orciprenaline | Anti-Asthmatic | Small Molecule | Approved | Agonist |
|  | [Metipranolol](file:////drugs/nhs/metipranolol%20) | Anti-Arrhythmia ; Antihypertensive ; Anti-Glaucoma | Small Molecule | Approved | Antagonist |
|  | [Nadolol](file:////drugs/nhs/nadolol%20) | Anti-Arrhythmia ; Antihypertensive | Small Molecule | Approved | Antagonist |
|  | [Nebivolol](file:////drugs/nhs/nebivolol%20) | Antihypertensive | Small Molecule | Approved | Antagonist |
|  | Norepinephrine | For Prevention Of Dermatitis During Radiotherapy | Small Molecule | Approved | Agonist |
|  | Norepinephrine | Antihypotensive ; Vasoconstrictor | Small Molecule | Approved | Agonist |
|  | [Olodaterol](file:////drugs/nhs/olodaterol%20) | Chronic Obstructive Pulmonary Disease (COPD) | Small Molecule | Approved | Agonist |
|  | [Oxprenolol](file:////drugs/nhs/oxprenolol%20) | Antihypertensive ; Anti-Arrhythmia | Small Molecule | Approved | Antagonist |
|  | Penbutolol | Antihypertensive | Small Molecule | Approved | Antagonist |
|  | [Pindolol](file:////drugs/nhs/pindolol%20) | Antihypertensive | Small Molecule | Approved | Partial Agonist |
|  | Pirbuterol | Obstructive Lung Disease | Small Molecule | Approved | Agonist |
|  | [Ritodrine](file:////drugs/nhs/ritodrine%20) | Tocolytic | Small Molecule | Approved | Agonist |
|  | [Salbutamol](file:////drugs/nhs/salbutamol%20) | Symptomatic Exophthalmos Associated With Thyroid-Related Eye Disease | Small Molecule | Approved | Agonist |
|  | [Salmeterol](file:////drugs/nhs/salmeterol%20) | Bronchodilator | Small Molecule | Approved | Agonist |
|  | [Salbutamol](file:////drugs/nhs/salbutamol%20) | Chronic Obstructive Pulmonary Disease (COPD) | Small Molecule | Approved | Agonist |
|  | [Salbutamol](file:////drugs/nhs/salbutamol%20) | Bronchodilator | Small Molecule | Approved | Agonist |
|  | [Sotalol](file:////drugs/nhs/sotalol%20) | Cardiovascular Disease | Small Molecule | Approved | Antagonist |
|  | [Terbutaline](file:////drugs/nhs/terbutaline%20) | Bronchodilator ; Tocolytic | Small Molecule | Approved | Agonist |
|  | [Timolol](file:////drugs/nhs/timolol%20) | Antiglaucomic | Small Molecule | Approved | Antagonist |
|  | [Vilanterol](file:////drugs/nhs/vilanterol%20) | Chronic Obstructive Pulmonary Disease (COPD) | Small Molecule | Approved | Agonist |
|  | [Carvedilol](file:////drugs/nhs/carvedilol%20) | Cardiovascular Disease | Small Molecule | Approved | Antagonist |
|  | Desipramine | Anti-Depressant | Small Molecule | Approved | Antagonist |
|  | Dobutamine | Heart Failure | Small Molecule | Approved | Agonist |
|  | Esmolol | Tinnitus | Small Molecule | Approved | Antagonist |
|  | Isoetharine | Obstructive Lung Disease | Small Molecule | Approved | Agonist |
|  | [Propafenone](file:////drugs/nhs/propafenone%20) | Atrial Fibrillation | Small Molecule | Approved | Antagonist |
|  | [Propranolol](file:////drugs/nhs/propranolol%20) | Antihypertensive | Small Molecule | Approved | Antagonist |
| FSH | Corifollitropin Alfa | Fertility | Protein | In Trial | Agonist |
|  | Fsh-Gex | Fertility | Protein | In Trial | Agonist |
|  | [Follitropin](file:////drugs/nhs/follitropin%20) | Fertility | Protein | Approved | Agonist |
|  | Menotropins, Menotrophin | Fertility | Protein | Approved | Agonist |
|  | [Urofollitropin](file:////drugs/nhs/urofollitropin%20) | Fertility | Protein | Approved | Agonist |
|  | Chorionic Gonadotropin | Fertility | Protein | Approved | Agonist |
| AGTR1 | Dsc127 | Diabetic Foot Ulcers | Peptide | In Trial | Agonist |
|  | Ljpc-501 | Hepatorenal Syndrome-Catecholamine-Resistant Hypotension (CRH) | Peptide | In Trial | Agonist |
|  | Ps433540 | Focal Segmental Glomerulosclerosis, Antihypertensive | Small Molecule | In Trial | Antagonist |
|  | Re-021, Sparsentan | Kidney Disease; Nephropathy | Small Molecule | In Trial | Antagonist |
|  | Saprisartan | Antihypertensive | Small Molecule | In Trial | Antagonist |
|  | Tasosartan | Antihypertensive | Small Molecule | In Trial | Antagonist |
|  | Trv120027 | Antihypertensive | Protein | In Trial | Antagonist |
|  | [Azilsartan Medoxomil](file:////drugs/nhs/azilsartan%20medoxomil%20) | Antihypertensive | Small Molecule | Approved | Antagonist |
|  | [Candesartan Cilexetil](file:////drugs/nhs/candesartan%20cilexetil%20) | Antihypertensive | Small Molecule | Approved | Antagonist |
|  | [Eprosartan](file:////drugs/nhs/eprosartan%20) | Antihypertensive | Small Molecule | Approved | Antagonist |
|  | Forasartan | Antihypertensive | Small Molecule | Approved | Antagonist |
|  | [Irbesartan](file:////drugs/nhs/irbesartan%20) | Antihypertensive | Small Molecule | Approved | Antagonist |
|  | [Losartan](file:////drugs/nhs/losartan%20) | Antihypertensive ; For Diabetic Neuropathy | Small Molecule | Approved | Antagonist |
|  | [Losartan](file:////drugs/nhs/losartan%20) | Antihypertensive | Small Molecule | Approved | Antagonist |
|  | [Olmesartan Medoxomil](file:////drugs/nhs/olmesartan%20medoxomil%20) | Antiglaucomic | Small Molecule | Approved | Antagonist |
|  | [Olmesartan Medoxomil](file:////drugs/nhs/olmesartan%20medoxomil%20) | Antihypertensive | Small Molecule | Approved | Antagonist |
|  | [Olmesartan Medoxomil](file:////drugs/nhs/olmesartan%20medoxomil%20) | Cardiovascular Disease | Small Molecule | Approved | Antagonist |
|  | [Telmisartan](file:////drugs/nhs/telmisartan%20) | Antihypertensive | Small Molecule | Approved | Antagonist |
|  | [Valsartan](file:////drugs/nhs/valsartan%20) | Antihypertensive | Small Molecule | Approved | Antagonist |
| GPER | MK-0354 | Atherosclerosis | Small Molecule | Investigational | Agonist |
|  | Genistein | Prostate cancer | Small Molecule | Investigational | Agonist |
|  | Estradiol | Atrophic Vaginitis  Breast Cancer  Breast engorgement caused by Postpartum state  Hypogonadism female  Kraurosis Vulvae  Metastatic Breast Cancer  Osteoporosis  Postmenopausal Osteoporosis  Premature Ovarian Failure (POF)  Prostate Cancer  Urogenital atrophy  Vasomotor Symptoms Associated With Menopause  Vulvo Vaginal Atrophy  Advanced androgen dependent Prostate cancer  Female castration  Hypoestrogenism  Contraception | Small Molecule | Approved | Agonist |

Source: GPCRdb (Kooistra et al., 2021)
